# Usefulness of Myocardial Deformation Indices in Preventing Cardiotoxicity in Breast Cancer Patients

**DOI:** 10.5935/abc.20190009

**Published:** 2019-01

**Authors:** Marcelo Dantas Tavares de Melo, Vera Maria Cury Salemi

**Affiliations:** Instituto do Coração (InCor) do Hospital das Clinicas da Faculdade de Medicina da Universidade de São Paulo, São Paulo, SP - Brazil

**Keywords:** Ventricular Dysfunction, Drug Therapy, Cardiotoxicity, Breast Cancer, Antineoplastic Agents

The first description of chemotherapy-induced heart failure (Stage C) was published in 1967.^1^ There has been a therapeutic evolution in oncological treatment since then, as shown by the fact that, as of 2005, the survival rate exceeded that of mortality.^2^ This has resulted in a new epidemiological problem for these survivors, since at least 30% of them will show some degree of cardiotoxicity, which can occur up to decades after the end of the chemotherapy. Moreover, cardiovascular mortality is already considered the second most common cause of death, second only to cancer.^3^^-^^5^

The classically accepted definition for cardiotoxicity during treatment was proposed in 2014, which described it as an absolute decrease in left ventricular (LV) ejection fraction of 10 percentage points to values below 53%, with its re-evaluation being recommended after 2 to 3 weeks. Additionally, the subclinical lesion is based on the relative decrease in global LV longitudinal strain by 15% in relation to the baseline.^6^ The major concern is that systolic dysfunction can lead to a therapeutic dose adjustment, less effective alternative therapy regimens, or, in the worst-case scenario, to chemotherapy discontinuation.

In 2016, the European Society of Cardiology reviewed the definition of chemotherapy-induced cardiotoxicity and extended it to include any structural or functional alteration in the heart and circulation, whether during cancer treatment, post-treatment or late post-treatment.^7^ That requires a conceptual amplification of the rationale in the cardiac monitoring of the oncological patient, which was previously restricted to an arbitrary ejection fraction value, without respecting the individualization of the patient’s hemodynamic parameters, gender and age, which all influence ejection fraction calculation.

It is important to note that the ejection fraction calculated by Simpson's two-dimensional method does not evaluate alterations in LV segmental contractility corresponding to 25% of its segments, considering the segmentation of 16 segments:^8^ the mid-basal portion of the inferolateral wall (two segments) and the mid-basal portion of the anteroseptal wall (two segments) are not analyzed, and this technical limitation is overcome by the three-dimensional echocardiogram.^9^ Considering this problem and a pragmatic observation of those who follow this patient population, the relevance of the isolated LV segmental alterations as chemotherapy-induced toxicity and its prognostic impact has been considered.

A case-control study published in 2017 showed that the segmental motility alteration in the interventricular septum was associated with a reduction in left ventricular performance, despite the presence of a preserved ejection fraction.^10^

The study published in this issue evaluated a prospective cohort of breast cancer patients and showed the incremental value of altered LV segmental motility in predicting cardiotoxicity induced by anthracyclines and/or trastuzumab.^11^ It is noteworthy that a high cardiotoxicity rate (16.1%) was observed in a population of which 35% were hypertensive; 22% were smokers; 19% were dyslipidemic and 7% were diabetics. There is no description in the present study of the doxorubicin and trastuzumab doses used in the treatment, the interval between examinations was variable between the groups, and whether the appearance of segmental motility alterations could be related to obstructive coronary disease, since several patients had risk factors.

Weberpals et al. in 2018^12^ described a cohort of 347,476 breast cancer patients exposed to chemotherapy or radiotherapy during a follow-up of more than 10 years and who showed no increase in cardiac mortality when compared to the general population.^12^

Another relevant piece of information not described in the text was whether there was a decrease of more than 15% of the LV global longitudinal strain (GLS) in patients who showed segmental contractility alterations. It is already well established that LV GLS is capable of predicting the reduction in LV ejection fraction^13^ and, in some institutions, it is indicated to initiate cardioprotection drugs even in the presence of a preserved ejection fraction. It is interesting to note that the segmental motility alterations described in 14% of the patients in the aforementioned article (interventricular septum, inferior and inferolateral) are the same regions that physiologically show coronary flow reduction.^14^

The proposed concept as one of the pathophysiological possibilities for the preferential segmental involvement described in Chagas' disease is that the terminal circulation - between the anterior descending coronary artery and the posterior descending artery (LV apex) and the terminal circulation between the right coronary artery and the left circumflex artery (the basal inferolateral segment) - contributes to the Chagasic lesion in these regions. Thus, it is likely that the aggressive agent (chemotherapy agent, or the *Trypanosoma cruzi*, for instance) would show a slower clearing in these regions, increasing the time of cardiomyocyte deleterious exposure.

Undeniably, chemotherapy-induced cardiotoxicity is multifactorial, but perhaps such a pathophysiological hypothesis might have a clinical consequence when endothelial and coronary vasomotor functions are improved prior to exposure to chemotherapy (statins, vasodilators, beta-blockers). Of the 14 patients with altered segmental contractility, 50% of cases consisted of atypical septal movement. Nevertheless, changes in septal movement constitute a nonspecific finding, as there is an extensive range of etiologies that alter septal motility, such as conditions that cause LV volume or pressure increase; primary involvement of the cardiomyocyte (cardiomyopathies); electric conduction changes; post-surgical status; pericardial disease; congenital cardiomyopathies; post-systolic shortening and interventricular mass^15^ and, therefore, one should be cautious in attributing such finding to cardiotoxicity, despite its plausibility.

An alternative that would help to understand the findings would be to expose the evolution of the LV GLS fall between the different groups and to analyze if there was any similarity between the findings of segmental alterations and the parametric arrangement of LV GLS. Although the importance of myocardial deformation segmental alterations is still debatable, there are studies that have shown the incremental role of this type of analysis.^16^^,^^17^

The present cohort described in the article mentioned in this editorial does not clarify how the groups were divided, making it difficult to understand how the statistical calculation was carried out. It would be interesting to have a univariate and a multivariate analysis of the factors that contributed to the ejection fraction decrease (systolic blood pressure, radiotherapy dose and site, chemotherapy dose, relative decrease in LV strain, initial absolute strain values, etc.). Moreover, a more detailed analysis of ventricular volumes and diastolic function would allow a better understanding of ventricular remodeling. Similarly, another limitation would be the inclusion of post-systolic shortening at the maximum strain peak, without considering the cardiac cycle phase.

Regardless of the exposed limitations, the article shows the relevance of a limited discussed finding, the alterations in LV segmental motility during chemotherapy treatment, which may be secondary to the disease, the treatment, or the decompensation of an underlying disease.
